# Synthetic Biology-Driven Microbial Therapeutics for Disease Treatment

**DOI:** 10.4014/jmb.2407.07004

**Published:** 2024-08-19

**Authors:** Tae Hyun Kim, Byung Kwan Cho, Dae-Hee Lee

**Affiliations:** 1 Synthetic Biology Research Center and the K-Biofoundry, Korea Research Institute of Bioscience and Biotechnology (KRIBB), Daejeon 34141, Republic of Korea; 2 Department of Biosystems and Bioengineering, KRIBB School of Biotechnology, University of Science and Technology (UST), Daejeon 34113, Republic of Korea; 3 Department of Biological Sciences, Korea Advanced Institute of Science and Technology (KAIST), Daejeon 34141, Republic of Korea; 4 KAIST Institutes for the BioCentury, KAIST, Daejeon 34141, Republic of Korea; 5 Graduate School of Engineering Biology, KAIST, Daejeon 34141, Republic of Korea; 6 Department of Integrative Biotechnology, College of Biotechnology and Bioengineering, Sungkyunkwan University, Suwon 16419, Republic of Korea

**Keywords:** Engineered microbial therapeutics, synthetic biology, GI disease, tumor, metabolic disease, infection

## Abstract

The human microbiome, consisting of microorganisms that coexist symbiotically with the body, impacts health from birth. Alterations in gut microbiota driven by factors such as diet and medication can contribute to diseases beyond the gut. Synthetic biology has paved the way for engineered microbial therapeutics, presenting promising treatments for a variety of conditions. Using genetically encoded biosensors and dynamic regulatory tools, engineered microbes can produce and deliver therapeutic agents, detect biomarkers, and manage diseases. This review organizes engineered microbial therapeutics by disease type, emphasizing innovative strategies and recent advancements. The scope of diseases includes gastrointestinal disorders, cancers, metabolic diseases, infections, and other ailments. Synthetic biology facilitates precise targeting and regulation, improving the efficacy and safety of these therapies. With promising results in animal models, engineered microbial therapeutics provide a novel alternative to traditional treatments, heralding a transformative era in diagnostics and treatment for numerous diseases.

## Introduction

The human microbiome, a community of microorganisms coexisting symbiotically with the human body, has been present since birth [1, 2]. Human microbiota colonizes all body sites, with the gastrointestinal (GI) tract harboring the highest microbial population [3]. Recently, it has been revealed that modifications to the microbial community within the gut caused by various factors such as diet, drugs, and environmental signals can lead to diseases in not only the intestine but also various organs [4]. Consequently, there is a diversification of efforts aimed at directly or indirectly restoring or maintaining a healthy gut microbiome to regulate human health [5].

With advancements in synthetic biology, the creation of microbes performing desired functions through the combination of bio-parts has become feasible. Particularly, research on engineered microbial therapeutics using probiotics or dominant strains as chassis is gaining attention as next-generation treatments [6-9]. Bacteria-based therapies offer advantages such as self-replication, the potential for diagnostic functions via genetic circuits, the ability for on-site production and delivery of therapeutic agents, omitting costly downstream processes, and reducing side effects [8]. Research in this area not only involves conferring roles as delivery vehicles for therapeutics or vaccines [10] but also encompasses more complex functions. These functions include detecting biomarkers of inflammation, disease, and pathogens based on genetically encoded biosensors, diagnosing [7, 9], and recording using memory devices [11, 12]. Additionally, to ensure the stability of these therapeutic agents, physical or biological containment (biocontainment) technologies are being developed to prevent exposure to bacteria after and during action [13, 14].

**Fig. 1:**
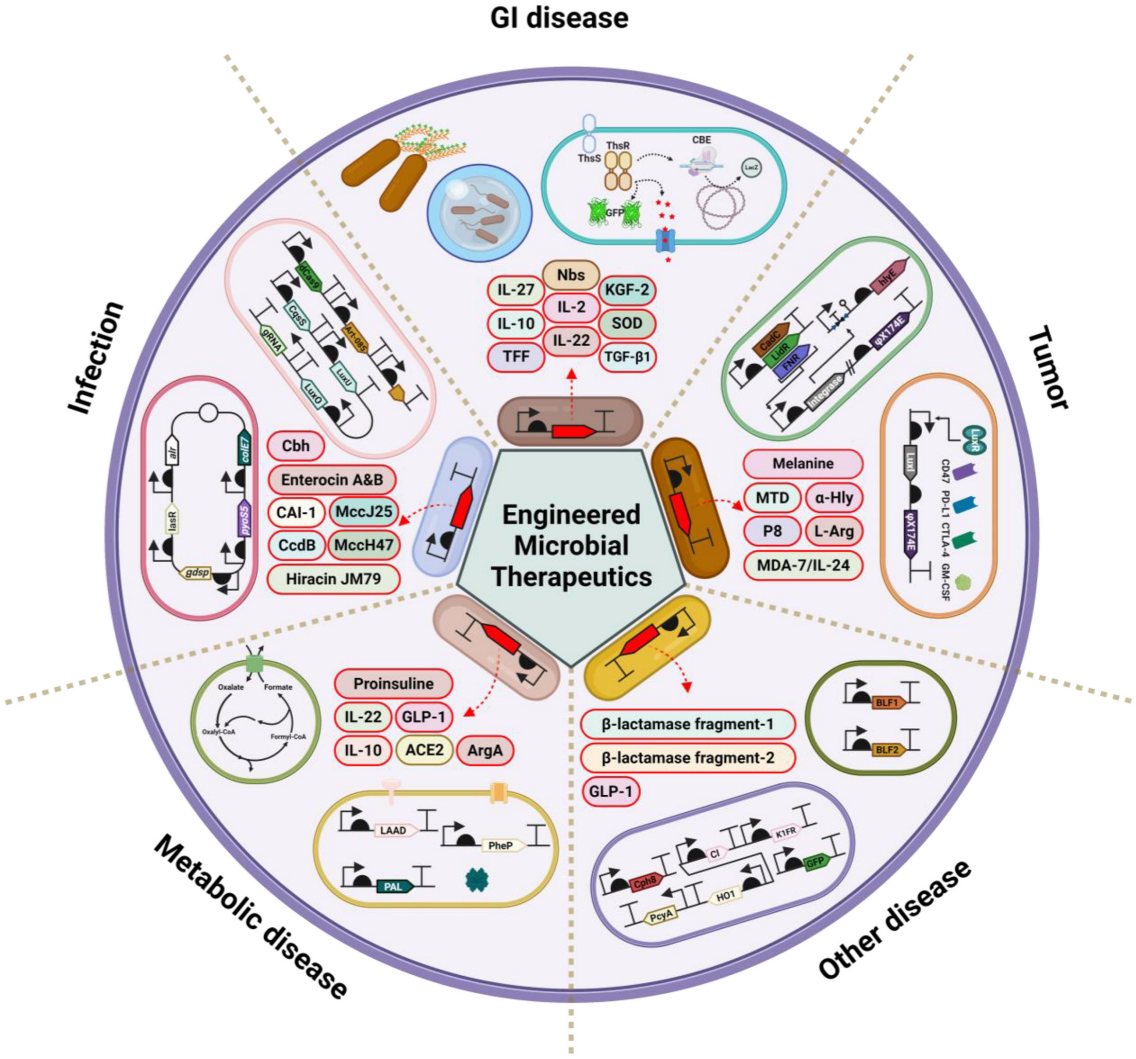
Schematic diagram of the utilization for engineered microbial therapeutics. Engineered microbial therapeutics, developed through synthetic biology, are applied to various conditions including gastrointestinal disease, tumor, pathogen infection, metabolic disease, and others. These applications utilize strategies that include not only simple production and delivery of therapeutic substances but also sophisticated regulatory mechanisms using genetic circuits for precise diagnosis, monitoring, and treatment.

In this review, engineered microbial therapeutics based on synthetic biology are classified according to disease (Fig. 1). Furthermore, each study is summarized and introduced regarding the strategies used including bio-parts, actuators and genetic circuits (Tables 1-5). Five representative categories are identified as actively researched areas up to the present: GI tract disease, tumors, metabolic disease, infectious disease, and others. This information will provide resources for researchers involved in engineered microbial therapeutics.

## Gastrointestinal Disease

Synthetic biology strategies for treating GI diseases have become increasingly diverse (Table 1). The GI tract is the primary focus for the application of engineered microbial therapeutics developed through synthetic biology, due to the feasibility of localized treatment. The prevalence of GI diseases is increasing, affecting nearly 2 million individuals in North America and Europe with chronic inflammatory disorders such as inflammatory bowel disease (IBD), which includes ulcerative colitis and Crohn’s disease [15-17].

### Encapsulation and Biocontainment

One notable approach involves the physical encapsulation of *Escherichia coli* Nissle 1917 (EcN), a potential therapeutic agent for intestinal diseases, within a chitosan-alginate matrix using layer-by-layer assembly and CaCl_2_ cross-linking. This method aims to ensure biocontainment and enhance survivability in the harsh environment of oral administration [14].

### Genetic Modifications for Enhanced Therapeutic Action

Several strategies involve direct modifications to EcN. For example, therapeutic curli hybrids have been designed by expressing trefoil factors fused to curli-forming proteins, promoting mucosal healing through matrix-tethered therapeutic domains [18]. Another approach targets ulcerative diseases by delivering epidermal growth factor using EcN, facilitating cell migration and extracellular matrix formation for intestinal damage repair [19]. Moreover, strategies to inhibit tumor necrosis factor-α (TNF-α) crucial target in IBD treatment - have been explored, including the use of nanobodies to neutralize TNF-α, with EcN’s type III secretion system enhancing nanobody delivery due to its gram-negative nature [20].

### Lactic Acid Bacteria (LAB) as Therapeutic Chassis

In addition to EcN, various strategies employ LAB as therapeutic chassis. These strategies primarily focus on protein secretion-based approaches, influencing the immune system directly or indirectly. Many target therapeutic molecules are interleukin (IL) family members, which have shown therapeutic efficacy in conditions like colitis [21-23]. For example, one study utilized *Lactococcus lactis* as a chassis to secrete IL-10 while knocking out the essential gene *thyA* to achieve auxotrophic biocontainment [13]. Other studies have enhanced wild-type *L. lactis* to secrete IL-27 to treat immune colitis [24], and genetically modified *Lactobacillus reuteri* to secrete IL-22 for conditions such as IBD and Graft versus Host Disease (GvHD) [25]. Furthermore, engineering efforts have increased IL-22 secretion by replacing the 2^nd^ and 17^th^ proline residues with glycine to counteract high proteolytic activity in *Lactobacillus*, preventing cleavage and enhancing IL-22 expression [26].

### Commensal Bacteria as Therapeutic Tools

Another research group utilized *Bacteroides ovatus*, a predominant gut commensal, as a chassis. They first engineered a system employing a xylan-inducible gene expression mechanism from the xylanase operon and a secretion signal sequence from the enterotoxin of *Bacteroides fragilis* to produce IL-2 [27]. They then modified this system to secrete trefoil factor-3 for validation purposes [28]. Further engineering enabled the secretion of transforming growth factor-β1 to evaluate therapeutic effects in a DSS-induced mouse model [29]. This was followed using the system to secrete keratinocyte growth factor 2, demonstrating its versatility [30]. The recent study showed improved secretion of heterologous therapeutic proteins in engineered *Bacteroides* species, particularly *Bacteroides thetaiotaomicron*, which is a significant component of the human gut microbiome [31]. The researchers identified novel signal peptides from *B. thetaiotaomicron* and *Akkermansia muciniphila* that enhance protein transport across cellular membranes. Additionally, they developed an episomal plasmid system that outperforms traditional chromosomal integration plasmids in protein secretion efficiency [31]. This plasmid system incorporates an essential gene (*thyA*)-based selection method to maintain plasmid stability without antibiotics, which is crucial for clinical applications. The study demonstrates the applicability of these advancements across multiple *Bacteroides* species, setting a new standard for developing live biotherapeutics aimed at treating gut diseases. Additionally, *Bifidobacterium longum*, known for its probiotic properties, was used as a chassis. In this study, a fusion protein of PEP-1 and manganese superoxide dismutase was expressed. This fusion protein was designed to convert reactive oxygen species, which increase during colitis onset, thus reducing intestinal inflammation [32].

### Intelligent Whole-cell Systems

In addition, there have been reports on systems designed not only for inflammation treatment but also for detecting inflammation biomarkers and releasing drugs only when these biomarkers are detected. In one such study, a genetic circuit was engineered into EcN to regulate the expression of several components: the expression of superfolder green fluorescent protein (sfGFP) for real-time signal confirmation through a bacterial two-component system capable of detecting thiosulfate as the selected biomarker, the induction of cytosine base editor along with sgRNA for inducing inheritable signals through base editing, and the expression of AvCystatin, an immunomodulatory protein. This intelligent whole-cell engineered bacteria system was designed to alleviate inflammation, demonstrating its potential effectiveness in inflammation relief [12]. A whole-cell biosensor using engineered EcN was reported to diagnose gut inflammation by sensing nitrate levels, a biomarker of inflammation [33]. The study employed the NarX-NarL two-component regulatory system to create a nitrate-responsive genetic circuit. This biosensor was optimized for sensitivity and specificity and successfully detected elevated nitrate levels in a mouse model of colitis. Additionally, they introduced a Boolean AND logic gate combining nitrate and thiosulfate sensing, enhancing the biosensor's specificity for gut inflammation [33].

### Chronic Inflammation Management

Chronic inflammation of the GI tract, the focus of these studies, is marked by recurrent cycles of symptom exacerbation and remission, with a multifactorial etiology. The complexity of its onset and progression often makes specific therapeutic interventions challenging, leading to the frequent use of systemic immunosuppressants, which carry significant risks of adverse effects [34]. The described research efforts continue to propose effective, less risky treatment modalities for managing the complexities of GI tract inflammation.

## Tumor

The application of engineered microbial therapeutics, enhanced through synthetic biology, is increasingly prominent in cancer treatment. Bacteria-based therapies for tumors offer several advantages, including their ability to colonize tumors and deliver therapeutic molecules directly to the site. This capability facilitates the direct killing of tumor cells and the induction of antitumor immune responses [35]. Several therapeutic strategies have emerged based on these advantages (Table 2).

### Facultative Anaerobes for Selective Colonization

Certain facultative anaerobes, known for their ability to selectively colonize tumors, have been utilized. One study modified *E. coli* χ6212 into an aspartate auxotroph by knocking out aspartate semialdehyde dehydrogenase (asd), allowing it to deliver pore-forming proteins from *S. aureus* to treat tumors [36]. Another strategy employed *E. coli* MG1655 as a chassis to secrete anti-tumor proteins via the type III secretion system from *Salmonella* [37].

### Immune System Modulation

EcN was engineered to produce cyclic di-AMP, a stimulator of interferon genes agonist, to induce type 1 interferon production and enhance anticancer effects [38]. This research also included strategies involving the knockout of essential genes for biocontainment and plasmid selection [38]. Indirect therapeutic enhancements have also been explored. For instance, EcN was engineered to overproduce L-arginine, which enhances the effectiveness of programmed death-ligand 1 (PD-L1) blocking antibodies, key immune checkpoint inhibitors [39]. Another approach used EcN to produce melanin, enhancing tumor photothermal and immunotherapy, and delivered via outer membrane vesicles coated with calcium phosphate for increased delivery efficiency [40].

### Targeted Tumor Recognition

Unique strategies for specific cancer types have been reported as well. For colorectal cancer (CRC), *Pediococcus pentosaceus* SL4 was engineered to produce P8, a protein from *Lactobacillus rhamnosus* CBT LR5, effectively reducing tumor volume and inhibiting growth [41, 42]. EcN was also designed to recognize the tumor microenvironment (TME) by incorporating sensors for lactate, pH, and hypoxia, leading to the secretion of hemolysin based on TME recognition [43]. Furthermore, a synthetic consortium of three bacterial strains was applied to treat CRC, reducing metabolic load and enhancing treatment efficacy [43].

### Enzyme Expression for Anticancer Compounds

Enzyme expression for converting dietary substances into therapeutic agents has also been investigated. In one study, EcN was engineered to express histone-like protein A on its surface to bind specifically to CRC cells and secrete an enzyme that converts glucosinolate from cruciferous plants into the anticancer compound sulforaphane near the tumor site [44]. For head and neck squamous cell carcinoma, *Bifidobacterium breve* was engineered to secrete melanoma differentiation-associated gene-7/interleukin-24 (MDA-7/IL-24), which induces apoptosis in various cancer cells [45-49].

### Innovative Bacterial Systems

Acyl homoserine lactone (AHL)-inducible sensors have been used to induce the production of therapeutic agents and cell lysis, facilitating therapeutic delivery. This system was initially used to design a strategy with CD47 antagonistic nanobodies, enhancing anti-tumor T cell priming by promoting phagocytosis of cancer cells and cross-presentation of tumor antigens [50]. Subsequently, therapies incorporating PD-L1 and CTLA-4 blocking nanobodies with granulocyte macrophage colony-stimulating factor production were developed [51]. This system was also applied to colorectal neoplasia, demonstrating therapeutic effects upon oral administration of engineered bacteria [52].

### Challenges and Future Directions

Bacterial cancer therapies represent a significant segment of the therapeutic market utilizing engineered microbes. Ongoing research continues to refine the strengths and address the weaknesses of commonly used bacterial strains, expand therapeutic strategies, and improve methods for delivering treatments [35].

## Metabolic Disease

Numerous studies have explored the treatment of metabolic diseases using engineered microbial therapeutics (Table 3). Metabolic diseases are conditions that disrupt the metabolic process, such as the conversion of food into energy at the cellular level. Diagnostic markers for these diseases involve the cellular ability to perform key biochemical reactions related to proteins (amino acids), carbohydrates (sugars and starches), or lipids (fatty acids)[53].

### Obesity

Obesity, a significant factor in metabolic diseases, often results from the excessive accumulation of triglycerides in adipose tissue [54]. Gut microbial diversity and composition also play a crucial role [55]. In this context, one strategy for treating obesity involves engineered *L. lactis* strains that secrete glucagon-like peptide-1 (GLP-1), a hormone that stimulates insulin secretion in a glucose-dependent manner. This approach enhances fatty acid oxidation, reduces blood triglyceride levels, and activates peroxisome proliferator-activated receptors α (PPARα) to improve liver tissue, thus alleviating obesity [54].

### Liver Disease

The liver is vital in lipid and glucose metabolism in obese individuals. Liver diseases, which result from abnormalities in these metabolic processes, are categorized based on alcohol influence. Non-alcoholic fatty liver disease occurs when fat comprises more than 5% of liver weight due to non-alcoholic factors [56], while alcoholic liver disease results from excessive ethanol intake, leading to hepatic steatosis, alcoholic steatohepatitis, and hepatocellular carcinoma [57]. IL-20 family cytokines, particularly IL-22, have shown potential in reducing liver injury through the activation of the STAT3 signaling pathway [58, 59]. Engineered *L. reuteri* strains secreting IL-22 have demonstrated therapeutic effects in both alcoholic and non-alcoholic fatty liver diseases [60, 61].

### Diabetes

Engineered microbes have also been utilized to treat diabetes, a prevalent metabolic disease. For autoimmune diabetes, *L. lactis* has been engineered to secrete human proinsulin and IL-10, which, when administered with low-dose anti-CD3 antibodies, reversed hyperglycemia and reduced insulin autoantibody levels, effectively reversing diabetes [62]. Additionally, in treating diabetic retinopathy, engineered *Lactobacillus paracasei* secreting angiotensin-converting enzyme 2 reduced inflammation and oxidative stress within the renin-angiotensin system, improving diabetic retinopathy outcomes [63].

### Addressing Genetic Metabolic Disorders

Therapeutic interventions have also targeted metabolic diseases caused by the loss of genes encoding metabolic enzymes. For example, enteric hyperoxaluria (EH) results from the accumulation and absorption of dietary oxalate in the intestines [64]. A strategy to address EH involves engineering EcN to express oxalate transporters and enzymes that convert oxalate into fumarate, reducing intestinal oxalate levels [65]. Similarly, hyperammonemia, a condition causing hepatic encephalopathy due to ammonia accumulation, has been addressed by altering the inhibitory feedback mechanism in EcN. By knocking out the repressor ArgR and expressing a feedback-resistant version of N-acetylglutamate synthase, this modification demonstrated therapeutic efficacy in suppressing hyperammonemia [66]. For phenylketonuria, characterized by neurotoxicity due to phenylalanine accumulation, engineered EcN was used to convert phenylalanine into non-toxic trans-cinnamate (TCA) while also expressing membrane-anchored L-amino acid deaminase to convert phenylalanine into phenyl pyruvate extracellularly [67]. Subsequent improvements involved selecting a more efficient phenylalanine ammonia lyase mutant using a TCA biosensor, resulting in a microbial therapy twice as effective as the previous version [68]. Further enhancements included creating genomic landing pads on EcN chromosomes for gene insertion, utilizing transcription factor-based sensors with small molecule ligands to improve phenylalanine conversion by 50% compared to existing methods [69]. Continuous advancements in using engineered microbes to express enzymes or substances that activate desired metabolic pathways highlight the potential for treating metabolic disorders.

## Infection

Infectious diseases remain a leading cause of mortality in clinical settings [70]. While antibiotic administration is a common treatment, it can eliminate beneficial microbes and promote antibiotic-resistant bacteria. Consequently, research on engineered microbes for treating infections is actively progressing (Table 4).

### Pseudomonas aeruginosa

To combat *P. aeruginosa* infections in the GI tract [71], researchers have explored two main strategies across three studies. The first strategy utilized sensing, lysing, and killing devices. Two studies employed *P. aeruginosa*'s type I quorum sensing mechanism to detect 3OC_12_HSL using a transcription factor-based sensor. Upon detection, these sensor cells expressed enzymes that caused self-lysis and released bacteriocins, resulting in the pathogen's destruction [72, 73]. The second strategy used the same biosensor but with a different output: secretion of DNaseI to break down biofilms, attraction of the pathogen through chemotaxis, and secretion of bacteriocins to kill the pathogen [74].

### Vibrio cholerae

For *V. cholerae*, which causes 21,000 to 143,000 deaths annually [75], treatment strategies vary based on the use of biosensors. Without biosensors, one approach involved engineering EcN to express both CAI-1 and AI-2 to suppress virulence gene expression in *V. cholerae* [76, 77]. Another strategy targeted antibiotic-resistant *V. cholerae* by using a type II bacterial toxin-antitoxin system as an actuator, which selectively killed the pathogen upon horizontal gene transfer of the plasmid [78]. With biosensors, a sensing, lysing, and killing device was developed. This device used a CAI-1 sensor to trigger cell lysis and release lysing enzymes to kill the pathogen [79].

### Salmonella enterica

To combat *S. enterica*, which causes significant foodborne illness [80, 81], strategies without biosensors involved engineering EcN strains to secrete antimicrobial peptides, achieving a 25-fold higher clearance rate in turkeys compared to controls [82]. Another approach used a tetrathionate sensor to regulate antimicrobial peptide expression, providing both bactericidal effects and resource competition [83].

### Clostridium difficile

For *C. difficile*, which thrives due to gut dysbiosis, researchers engineered a sensor for sialic acid, which increases during dysbiosis [84]. This sensor regulated enzymes converting taurocholate, a *C. difficile* germination inducer, into cholate, thus preventing infection by modulating germination [85].

### *Enterococci* and *Candida albicans*

Additionally, strategies to inhibit *Enterococci* growth, which causes nosocomial infections and shows antibiotic resistance, involved using three antimicrobial peptides simultaneously [86]. Research targeting *C. albicans*, a fungus causing opportunistic infections, focused on identifying and detecting secreted substances using biosensors to inhibit hypha formation, a virulence factor [87].

### Ongoing Research and Future Directions

Various therapeutic strategies are being explored to replace antibiotics with engineered bacterial therapeutics, aiming to combat infections caused by bacteria and fungi more safely and effectively.

## Other Diseases

In addition to GI diseases, tumor, and metabolic diseases, synthetic biology and engineered microbial therapeutics offer promising solutions for a range of other diseases. These approaches leverage the precision of genetic circuits and the versatility of microbial systems to address complex health challenges (Table 5).

### Neurodegenerative Diseases

One significant area of application is neurodegenerative diseases, particularly through the regulation of the gut-brain axis. Two notable studies focus on treating Parkinson’s disease (PD), characterized by the loss of dopaminergic neurons in the brain. Both studies utilized GLP-1 and its analog Exendin-4 as therapeutic agents due to their known beneficial effects on PD [88]. GLP-1 can cross the blood-brain barrier, protect neurons from oxidative stress-induced apoptosis, and promote neuronal proliferation, making it a potential treatment [89, 90]. In one study, *L. lactis* was engineered to continuously express and secrete GLP-1 [91]. Another study, engineered a strain to constitutively express Exendin-4 and incorporated a red-light inducible chimeric light sensor to regulate the expression of a cell lysis enzyme, allowing controlled drug release through red-light stimulation with high skin penetrability [92]. These studies highlight the potential for treating diseases in organs beyond the gut through diverse therapeutic production and delivery methods.

### Antibiotic-induced Dysbiosis

Another area of research targets antibiotic-induced dysbiosis. One study focused on β-lactamase and employed *L. lactis* as a chassis, splitting β-lactamase into two fragments fused with SpyTag/Catcher for extracellular secretion [93]. Unlike β-lactamase in Gram-negative bacteria, which is located in the periplasm and confers antibiotic resistance to single cells [94], this study used Gram-positive bacteria, making survival dependent on cell density. When the engineered microbes and antibiotics were administered, the gut microbiota exhibited less disruption compared to the control group, demonstrating an inhibitory effect on pathogenic strains such as *C. difficile* [93]. This research exemplifies the broader therapeutic applications of engineered microbes.

Indeed, synthetic biology-driven engineered bacterial therapeutics hold significant potential for targeting various organs and disease targets, contingent upon the identification and combination of appropriate bio-parts as demonstrated in these studies.

## Challenges and Future Directions

While engineered microbial therapeutics hold significant promise, several challenges must be addressed to bring these treatments from the lab to the clinic. For GI diseases, one major hurdle is ensuring the stability and survivability of therapeutic microbes in the harsh conditions of the GI tract. Approaches like encapsulation [14] and biocontainment [13, 95, 96] are being developed to overcome these challenges, but further research is needed to ensure consistent efficacy in human trials. In the case of cancers, the primary challenge lies in the selective targeting of tumors [44] and minimizing off-target effects [44, 97]. Strategies such as engineering microbes to respond to tumor-specific biomarkers are promising but require rigorous validation. Metabolic diseases present a unique challenge in achieving sustained therapeutic effects without disrupting the body's metabolic balance [98]. Finally, for infectious diseases, the main obstacle is developing therapies that are potent enough to eradicate pathogens without promoting antibiotic resistance or disrupting the native microbiota [73].

To gain approval as new drugs, engineered microbial therapeutics must overcome significant regulatory hurdles. These include demonstrating safety and efficacy through preclinical studies and clinical trials. Additionally, regulatory agencies require comprehensive data on the genetic stability and biocontainment of these organisms to prevent unintended environmental release [95]. Establishing standardized protocols for the production and quality control of these biotherapeutics is also crucial. Collaborative efforts between researchers, regulatory bodies, and industry stakeholders will be essential to streamline this process. To create safe and effective engineered microbial therapeutics, several standards and genetic methods should be applied. These include genetic stability, rigorous testing, standardization, and regulatory compliance. To ensure the safety and efficacy of engineered microbial therapeutics, several critical measures must be taken. First, incorporating genetic safeguards such as kill switches and biocontainment mechanisms is essential to prevent horizontal gene transfer and environmental persistence [14, 92, 99, 100], thereby ensuring genetic stability. Rigorous testing, including extensive in vitro and *in vivo* studies, is necessary to evaluate the long-term safety and therapeutic efficacy of these microbes. Developing standardized protocols for genetic modifications and therapeutic production is crucial to maintain consistency across different batches and studies. Finally, adherence to regulatory guidelines set by agencies such as the FDA and EMA is imperative. This includes submitting detailed data on genetic constructs, production methods, and safety evaluations to comply with regulatory requirements. By implementing these measures, the development of safe and effective microbial therapeutics can be achieved.

The use of live genetically modified microorganisms presents several challenges, including environmental exposure, phenotypic changes, and non-specific mutations. To mitigate these issues, several strategies must be employed. First, physical and genetic biocontainment strategies are crucial to prevent the release and spread of modified organisms in the environment [96]. Ensuring the phenotypic stability of therapeutic strains through rigorous genetic and phenotypic screening is essential. Advanced gene editing techniques should be used to minimize off-target effects and non-specific mutations. Additionally, designing therapeutics that do not rely on antibiotic resistance markers and developing alternative selection methods can help manage antibiotic resistance [13, 31]. Finally, conducting detailed pharmacokinetic studies is important to understand the distribution, persistence, and clearance of these therapeutics in the human body. By addressing these challenges, the safe and effective use of live genetically modified microorganisms can be achieved.

## Conclusion

Looking ahead, several key areas should be prioritized to further advance the field of synthetic biology and engineered microbial therapeutics. First, there is a need for continued development and refinement of genetic circuits that can provide more precise and robust control over microbial functions. This includes the creation of more sophisticated biosensors and actuators that can respond to a wider range of biological signals and environmental conditions. Additionally, enhancing the stability and safety of these engineered microbes is crucial, particularly through the implementation of biocontainment strategies that prevent unintended spread and ensure that therapeutic functions are restricted to the target site. Another important direction is the integration of engineered microbial therapeutics with other treatment modalities, such as conventional drugs, immunotherapies, and personalized medicine approaches. Combining these therapies can enhance their effectiveness and reduce the likelihood of resistance. For example, engineered bacteria that deliver checkpoint inhibitors directly to tumor sites could complement systemic immunotherapies, providing a more targeted and potent anti-cancer strategy. Moreover, advancements in genome editing technologies, such as CRISPR-Cas systems, can facilitate the development of more precise and efficient microbial therapeutics, enabling the correction of genetic disorders and the modulation of complex metabolic pathways [101, 102]. Finally, rigorous clinical evaluation and regulatory frameworks are essential to translate the promising research findings into safe and effective therapies for patients. This includes conducting well-designed clinical trials to assess the efficacy, safety, and long-term effects of engineered microbial therapeutics in diverse patient populations. Collaboration between researchers, clinicians, regulatory agencies, and industry stakeholders will be critical to overcoming the challenges and accelerating the adoption of these innovative therapies in clinical practice. In conclusion, advancements in synthetic biology and engineered microbial therapeutics hold great promise for revolutionizing the treatment of a wide array of diseases. By continuing to innovate and address the remaining challenges, we can unlock the full potential of these cutting-edge therapies and improve the health and well-being of patients worldwide.

## Figures and Tables

**Table 1:** Reports on engineered bacterial therapeutics for GI disease.

Chassis	Target disease	Genetically encoded biosensor	Actuator	Model	Ref.
*E. coli* Nissle 1917	IBD	-	Encapsulated microbe	Sprague-Dawley rat	[14]
*E. coli* Nissle 1917	Colitis	-	Curli-fused trefoil factor	C57BL/6NCrl mice	[18]
*E. coli* Nissle 1917	Intestinal ulcerative disease	-	Epidermal growth factor	C57BL/6 mice	[19]
*E. coli* Nissle 1917 Δ*thyA*, *alr*	IBD	-	Type3 secretion apparatus TNF-α neutralizing nanobody	C57BL/6 mice BALB/c mice	[20]
*Lactococcus lactis* Δ*thyA*	IBD	-	Interleukin-10	Piétrain/Landrace crossbred pigs	[13]
*L. lactis*	Colitis	-	Interleukin-27	C57BL/6 mice	[24]
*Lactobacillus reuteri*	Intestinal disease (GvHD, IBD)	-	Interleukin-22	-	[25]
*Bacteroides ovatus*	Chronic gut disorder	-	Interleukin-2	-	[27]
*B. ovatus*	Chronic gut disorder	-	Trefoil factor	-	[28]
*B. ovatus*	Colitis	-	Transforming growth factor-β1	C57BL/6 mice	[29]
*B. ovatus*	Colitis	-	Keratinocyte growth factor-2	C57BL/6 mice	[30]
*Bifidobacterium longum*	Colitis	-	PEP-1 fused manganese superoxide dismutase	Sprague-Dawley rat	[32]
*E. coli* Nissle 1917	IBD	Thiosulfate-inducible sensor for the sensing of biomarker and regulation of genes expression	Cytosine base editor sgRNA sfGFP Hly fused AvCystatin	C57BL/6J mice	[12]

**Table 2:** Reports on engineered bacterial therapeutics for tumor.

Chassis	Target disease	Genetically encoded biosensor	Actuator	Model	Refs
*E. coli* χ6212 Δasd	Tumor	-	α-hemolysin	BALB/c mice	[36]
*E. coli* MG1655	Tumor	-	Type 3 secretion system Mitochondrial targeting domain of Noxa	BALB/c mice	[37]
*E. coli* Nissle 1917 Δ*dapA*, *thyA*	Tumor	-	Cyclic di-AMP	C57BL/6 mice BALB/c mice	[38]
*E. coli* Nissle 1917 Δ*argR*, *malEK*::*argA^fbr^*	Tumor	-	L-Arginine	C57BL/6 mice	[39]
*E. coli* Nissle 1917	Tumor	-	Melanine	BALB/c mice	[40]
*Pediococcus pentosaceus* SL4 Δ*alr*	Colorectal cancer	-	P8	BALB/c mice C57BL/6J mice	[42]
*E. coli* Nissle 1917	Colorectal cancer	Lactate, pH, Hypoxia-inducible sensor for the expression of serine integrase	Hemolysin	BALB/c mice C57BL/6J mice	[43]
*E. coli* Nissle 1917	Colorectal cancer	-	INP-tagged histone-like protein A YebF-fused I1 myrosinase	BALB/c mice	[44]
*Bifidobacterium breve*	Head and neck squamous cell carcinoma	-	MDA-7/IL-24	BALB/c mice	[49]
*E. coli* Pir1^+^	Tumor	AHL-inducible sensor for the production of transcription factor and lysis protein	Phage-lysis protein (ϕX174E) HA-tagged CD47 antagonistic nanobody	BALB/c mice C57BL/6 mice	[50]
*E. coli* Nissle 1917	Tumor		Phage-lysis protein (ϕX174E) HA-tagged PD-L1 blocking nanobody HA-tagged CTLA4 blocking nanobody Granulocyte-macrophage colony-stimulation factor	BALB/c mice	[51]
*E. coli* Nissle 1917 Δ*clbA*	Colorectal neoplasia			C57BL/6 mice	[52]

**Table 3:** Reports on engineered bacterial therapeutics for metabolic disorder.

Chassis	Target disease	Genetically encoded biosensor	Actuator	Model	Refs
*L. lactis*	Obesity	-	GLP-1	C57BL/6 mice	[54]
*L. reuteri*	Nonalcoholic fatty liver disease	-	Interleukin-22	C57BL/6J mice	[60]
*L. reuteri*	Alcoholic liver disease	-	Interleukin-22	C57BL/6 mice	[61]
*L. lactis*	Diabetes	-	Proinsulin Interleukin-10	NOD mice	[62]
*Lactobacillus paracasei*	Diabetic retinopathy	-	Angiotensin converting enzyme 2	C57BL/6J mice	[63]
*E. coli* Nissle 1917 Δ*thyA*	Enteric hyperoxaluria	-	Oxalate/formate antiporter Oxalyl-CoA decarboxylase Formyl-CoA trasferase	C57BL/6J mice Cynomolgus monkey	[65]
*E. coli* Nissle 1917 Δ*thyA*, *argR*	Hepatic encephalopathy	-	N-acetylglutamate synthase	C57BL/6 mice Cynomolgus monkey Human (healthy)	[66]
*E. coli* Nissle 1917 Δ*dapA*	Phenylketonuria	-	Phenylalanine transporter Phenylalanine ammonia lyase L-amino acid deaminase	C57BL/6 mice Cynomolgus monkey	[67]
		Trans-cinnamate-inducible sensor for the screening of phenylalanine ammonia lyase activity	Phenylalanine transporter Phenylalanine ammonia lyase mutant L-amino acid deaminase	Cynomolgus monkey	[68]
		Small molecule-inducible sensor array for the regulation of enzyme expression		-	[69]

**Table 4:** Reports on engineered bacterial therapeutics for infection.

Chassis	Pathogen	Genetically encoded biosensor	Actuator	Model	Refs
*E. coli* TOP10	*Pseudomonas aeruginosa*	3OC_12_HSL-inducible sensor for the production of cell lysis enzyme and bacteriocin	Pyocin S5 E7 lysis protein	-	[72]
*E. coli* Nissle 1917 Δ*alr*, *dadX*		3OC_12_HSL-inducible sensor for the production of cell lysis enzyme, bacteriocin, and anti-biofilm enzyme	Pyocin S5 E7 lysis protein Dispersin B	Caenorhabditis elegans ICR mice	[74]
*E. coli* UU2685		3OC_12_HSL-inducible sensor for the activation of motility and killing modules	Microcin S DNaseI CheZ fused degron	-	[73]
*E. coli* Nissle 1917	*Vibrio cholera*	-	Cholera autoinducer 1	CD-1 mice	[77]
*E. coli* XL2 Blue		-	Type II gyrase inhibiting toxin	-	[78]
*E. coli* MG1655		Cholera autoinducer 1-inducible sensor for expression of lysis enzyme	Artilysin YebF fused Artilysin	Zebrafish larvae Crustacean larvae	[79]
*E. coli* Nissle 1917	*Salmonella enterica*	-	Microcin J25	Turkey	[82]
*E. coli* Nissle 1917		Tetrathionate-inducible sensor for the production of antimicrobial peptide and competition of resource	Microcin H47	-	[83]
*E. coli* Nissle 1917 Δ*alr*, *dadX*	*Clostiridium difficile*	Sialic acid-inducible sensor for diagnosis of antibiotic-induced dysbiosis	Bile salt hydrolase	C57BL/6 mice	[85]
*E. coli* Nissle 1917	*Enterococcus faecium* *Enterococcus faecalis*	-	Enterocin A Enterocin B Hiracin JM79	Balb/cJ mice	[86]
*E. coli* NGF-1	*Candida albicans*	Hydroxyphenylacetic acid-inducible sensor for the sensing of fungus	Cis-2-dodecenoic acid	-	[87]

**Table 5:** Reports on engineered bacterial therapeutics for other diseases.

Chassis	Target disease	Genetically encoded biosensor	Actuator	Model	Refs
*L. lactis*	Parkinson’s disease	-	GLP-1	C57BL/6 mice	[91]
*E. coli* Nissle 1917	Parkinson’s disease	Red-light inducible chimeric light sensor for production of cell lysis enzyme	Exendin-4 fused anti-neonatal FC receptor affibody	C57BL/6 mice	[92]
*L. lactis*	Antibiotic-induced dysbiosis	-	SpyTag fused β-lactamase fragment 1 SpyCatcher fused β-lactamase fragment 2	C57BL/6 mice	[93]
